# Implementing calculated globulin screening for immunodeficiencies: The benefits and challenges

**DOI:** 10.70962/jhi.20260028

**Published:** 2026-07-16

**Authors:** Stephen Jolles, Cecilia Poli, Nicholas L. Rider, Gabriela Negritu, Til Rendschmidt, Antonio Condino-Neto

**Affiliations:** 1 Immunodeficiency Centre for Wales, University Hospital of Wales, Cardiff, UK; 2 Program of Immunogenetics and Translational Immunology, Faculty of Medicine, Clínica Alemana Universidad del Desarrollo, Santiago, Chile; 3 Department of Health Systems & Implementation Science Virginia Tech Carilion School of Medicine, Department of Pediatrics, Section of Allergy-Immunology, The Carilion Clinic, Roanoke, VA, USA; 4 CSL AG, Bern, Switzerland; 5 CSL GmbH, Marburg, Germany; 6 Jeffrey Modell Diagnostic and Research Center, São Paulo, Brazil

## Abstract

There is a need for pragmatic and cost-effective methodologies to facilitate earlier diagnosis of patients with primary and secondary immunodeficiencies, who often experience diagnostic delays that impact their quality of life, morbidity, and mortality. This review article will consider the utility of calculated globulin (CG) as a simple and unbiased screening tool for immunodeficiencies. We review literature indicating that CG can serve as an inexpensive and routinely performed proxy for immunoglobulin levels due to their established correlation. We also set out a workflow for the implementation of CG into clinical practice and discuss optimal approaches to embedding into laboratory reporting systems, and integration of AI approaches could expedite diagnoses. Finally, we discuss the challenges and pitfalls of CG screening, including the need for established CG cutoffs/action values across different patient age cohorts. Collaboration among biochemistry and immunology laboratories, clinicians, and patient organizations will be required to manage the logistics of effective broader implementation of CG screening into clinical practice.

## Teaser

This review article considers the utility of calculated globulin as an unbiased and inexpensive screening tool for immunodeficiency. The benefits and practicalities of its clinical implementation and current challenges and limitations are discussed.

## Introduction

Primary immunodeficiencies (PIDs), also referred to as (part of) inborn errors of immunity (IEI), are a heterogenous group of disorders that affect ∼1 to 6 in 10,000 individuals (1, 2). Secondary immunodeficiencies (SID) encompass any dysfunction of the immune system resulting from extrinsic influences, including transplantation, protein loss, hematologic malignancies, some infections, and side effects of certain medications, to name a few (3, 4). Antibody deficiencies are characterized by reduced immunoglobulin (Ig) levels and are types of PID and SID (5). Whilst primary antibody deficiencies (PAD) comprise the largest group within PID (1, 5), secondary antibody deficiencies (SAD) are ∼30 times more common than PAD (4).

Hypogammaglobulinemia, which can be present in both PID and SID, is defined as a low concentration of circulating Igs and is associated with an increased risk of chronic, recurrent, and severe infections (6, 7). Although the reference range may vary across laboratories, hypogammaglobulinemia in adult patients is often defined by IgG levels below 5–7 g/L (6, 8).

### Diagnostic delay and the need to reduce it

Diagnostic delay can occur when there is a lack of awareness of immunodeficiencies (9) and/or a failure to consider immunodeficiency as part of the differential diagnosis in patients who present with the appropriate signs/symptoms (10, 11). Failure to consider PID is at least partly due to the misconception that PID only presents in childhood (11). The onset of common variable immunodeficiency (CVID), the most common PAD found in adults (12), has been reported in patients 3 to 79 years of age, with a median age of onset of 23 and 28 years in males and females, respectively (11). Patients with hematologic malignancies or autoimmune/inflammatory disorders may experience a delay in diagnosis of SAD and appropriate intervention, as the therapies and attention often focus on the underlying disease rather than potential antibody deficiency (4). Despite guidelines supporting the use of IgG levels to both identify and monitor patients with immunodeficiencies (13, 14, 15), real-world data indicate that IgG testing is not implemented consistently in routine clinical practice. A physician survey (*n* = 100) conducted in the US identified disparities among physician specialties regarding the frequency and parameters in which they would implement IgG testing in patients with chronic lymphocytic leukemia (CLL) and SID (Ramakrishna, B., P. Michaela, E. Gabriela, Z. Xiang, M. Rajiv, I. Fidelia, G. Leanne, Y. Lara, D. Elizabeth, S. John, et al. 2024. Secondary immunodeficiency in patients with chronic lymphocytic leukaemia: US physician and patient perspectives on infections and immunoglobulin replacement therapy use. 21st ESID Biennial Meeting.). Furthermore, in an analysis of real-world data of patients with CLL and SID (*n* = 2,898) in the US, IgG levels were rarely monitored in patients who had experienced a severe bacterial infection (Ramakrishna, B., G. Espinoza, X. Zhang, F. Ida, L. Yoon, J. Shen, D. Gardiner, and Q. Ataher. 2024. Real-world analysis of diagnostic patterns of severe bacterial infections in patients with chronic lymphocytic leukaemia and secondary immunodeficiency. 21st ESID Biennial Meeting.). Delayed diagnosis is a major issue for patients with rare diseases, particularly PID, and is associated with higher patient morbidity, including significant end-organ damage, permanent functional impairment, and higher mortality rates (10, 11, 16, 17). Patient-reported perceptions of well-being and health status are significantly improved in patients who experience a shorter time between symptom onset and diagnosis, compared with those who experience diagnostic delay (18).

A delayed diagnosis of PID/SID also results in a significant burden on healthcare and financial resources. In an evaluation of the annual cost of CVID that included direct, indirect, and intangible costs, early diagnosis was associated with savings of approximately US$6,500 per patient per year (19). When one considers that the median delay in diagnosis is 2 to 9 years (10, 11, 17), the financial cost alone of a delayed diagnosis of PID/SID becomes more stark (Alfego, D., et al. 2025. *J. Hum. Immun.* Abstract 204. https://doi.org/10.70962/cis2025abstract.204).

The lack of availability of laboratories and immunologists to carry out the specialized tests required once a patient has been identified as having hypogammaglobulinemia (based on IgG levels) can also contribute to a delayed immunodeficiency diagnosis (9). For example, in Brazil everyone is guaranteed access to the public health system, including basic laboratory tests such as IgG levels; however, only two of the five regions of Brazil are able to carry out more specialized tests for immunodeficiencies (9). A similar situation is observed in other countries in Latin America and other low- and middle-income countries, where overall availability of essential diagnostic tests is found to be lacking primarily due to high costs and a deficit in infrastructure and expertise (20, 21).

### Approaches for reducing diagnostic delay

Immunology laboratory assessments together with a careful review of a patient’s infection/microbiological history are key to assessing the impact of an antibody deficiency (e.g., hypogammaglobulinemia) as well as guiding appropriate treatment decisions, e.g., exposure modifications, antibiotics, vaccination, and Ig replacement therapy (IgRT).

International clinician-education initiatives, led by organizations such as the Jeffrey Modell Foundation, immunology societies, and patient groups, aim to improve awareness, interdisciplinary collaboration, and earlier diagnosis of immunodeficiencies through structured training, diagnostic guidance, congresses, and continuing medical education (CME) worldwide (2, 9, 22, 23, 24, 25, 26, 27, 28, 29, 30, 31).

Artificial intelligence (AI)–based diagnostic approaches, including International Classification of Diseases (ICD) code–driven tools and machine-learning algorithms applied to electronic health records, have demonstrated the ability to identify patients with immunodeficiencies earlier than standard clinical pathways, substantially reducing diagnostic delay (32, 33, 34, 35). However, their implementation is constrained by data availability and quality, variability in coding practices across healthcare systems, overlapping disease phenotypes, and bias within training datasets. Additional challenges include ethical considerations, data protection requirements, and the need for transparency and open science to support broader adoption (33, 36).

### Rationale for additional screening

Newborn screening (NBS) enables early detection of various treatable PIDs, such as severe combined immunodeficiency in asymptomatic infants and has been implemented in over 35 countries, primarily using T cell receptor excision circle (TREC) detection (37, 38, 39, 40, 41, 42, 43). In some regions, simultaneous κ-deleting recombination excision circle (KREC) detection (TREC/KREC assays) screening is used to identify early B cell disorders, including X-linked agammaglobulinemia (42, 44).

Although global implementation of NBS is a desirable goal, worldwide availability is still limited. Moreover, the growing use of immune suppressors is creating a growing patient population that may be at increased risk of SIDs that will not be detected by NBS (4, 45, 46, 47, 48). Furthermore, NBS will not detect antibody deficiencies such as CVID, due to its onset in later life and the presence of B and T cells (38, 49). Besides NBS, current methods to identify patients with immunodeficiencies are based on clinical events that have already happened (e.g., infections, bronchiectasis). Since early diagnosis is essential to prevent the onset of potentially irreversible clinical events, there is an unmet need for additional methodologies.

IgG levels conclusively identify patients with hypogammaglobulinemia, but the clinician must first suspect an antibody deficiency to prompt them to order that test—a challenge for many healthcare systems that are already overstretched. This underscores the urgent need for a pragmatic, repeatable, and cost-effective screening method to reduce diagnostic delay and improve clinical outcomes in patients with antibody deficiencies. This screening test should be simple to carry out, and the results should be easily understood by physicians across a broad range of specialties, with clear next steps should a patient’s test results indicate a possible immunodeficiency. Calculated globulin (CG) has the potential to fulfill these requirements.

The use of CG as a screening tool for PID and SID in adult and pediatric patients will be considered in this review, based on data from a growing number of global clinical studies across a range of healthcare settings. Practical guidance will also be provided to implement CG screening in routine clinical practice to help reduce delays in diagnosing immunodeficiencies worldwide.

## CG testing for immunodeficiency screening

CG (total protein minus albumin) is a standard test that commonly forms part of the liver function test (LFT) profile (17). LFTs are carried out at high frequency in routine clinical practice in both primary and secondary care settings (5, 50, 51). Total protein is measured using the Biuret method. Albumin can be measured using a colorimetric assay using either bromocresol green (BCG) or bromocresol purple (BCP) (13); the main differences between these assays relate to specificity and sensitivity, particularly at lower albumin concentrations (17).

CG measures the serum globulin concentration, of which Igs are the major component (17). CG levels are linearly associated with IgG levels (17, 52; Alfego, D., et al. 2025. *J. Hum. Immun.* Abstract 204. https://doi.org/10.70962/cis2025abstract.204), as well as with total Ig levels (IgG + IgA + IgM) in adults (10). Low CG levels have been shown to correlate with low IgG levels, which are indicative of hypogammaglobulinemia in adults, with high specificity and sensitivity in some studies and low sensitivity in others (Table 1) (5, 10, 11, 17, 50). Variation in specificity and sensitivity across studies likely reflects differences in patient cohorts and testing settings, rather than the calculation itself. In primary care, patients are typically more stable, so albumin and total protein more closely reflect baseline physiology and low CG more reliably tracks low Ig levels (5, 11, 17, 50). In secondary care and hospital cohorts, acute illness, inflammation, and comorbidity more often alter albumin and non-Ig proteins: acute-phase responses can raise the globulin fraction and mask low IgG (reducing sensitivity), while hemodilution (e.g., intravenous [IV] fluids during surgery or intensive care) can lower albumin/total protein and artifactually lower CG (reducing specificity) (10). High CG levels have been used widely in primary and secondary care to indicate the possibility of a hematologic malignancy (such as multiple myeloma) and may also indicate a range of high CG-related diseases, including autoimmune diseases and infections (17).

**Table 1.** Accuracy and sensitivity of CG cut-off values to identify hypogammaglobulinemia in adults

Country	CG action/cut-off levels (g/L)	Corresponding IgG level (g/L)	Sensitivity^a^	Specificity
**Albumin measured using BCG**				
Wales (*n* = 50) (17)	<18	<6	82%	71%
Italy (*n* = 25) (5)	<19	<6	70%	75%
Turkey (*n* = 50) (50)	<20	<6	84%	75%
Australia (*n* = 4,381) (10)	≤20	≤5	19%	99%
**Albumin measured using BCP**				
Wales (*n* = 50) (17)	<23	<5	77%	65%

IgG, immunoglobulin G; *n*, number of samples; BCG, bromocresol green; BCP, bromocresol purple; CG, calculated globulin.

a Variation in sensitivities among studies may correspond to the types of patient cohorts being tested (e.g., primary vs. secondary care patients) and subsequent rates of illnesses affecting the production and concentrations of albumin and immunoglobulins (10).

As CG is a measure of globulin quantity rather than quality (function; see Limitations), when there is a high index of suspicion of immunodeficiency (outside of an opportunistic screening setting), physicians should consider follow-on confirmatory tests, including IgG levels, functional antibodies, and vaccine responses.

### Validating the clinical utility of CG screening

While there is no universal standardized cut-off level for CG to identify patients with immunodeficiency, several global clinical studies have validated the utility of CG screening in adults and defined population-specific low levels as <18–20 g/L (using BCG to measure albumin) to identify patients with immunodeficiency (5, 17, 50, 52; Alfego, D., et al. 2025. *J. Hum. Immun.* Abstract 204. https://doi.org/10.70962/cis2025abstract.204; Jolles, S., and C. Poli, personal communication). The utility of CG screening is also demonstrated in three case studies of patients who were identified as having an underlying PID (namely CVID) after being referred to immunology following CG screening, who were then managed successfully using IgRT (17). In the experience of the author, Professor Jolles from University Hospital Wales, a major route of referrals for the service is now via CG screening. In addition, data from Labcorp presented at the 2025 Clinical Immunology Society (CIS) Annual Meeting demonstrated that 518,033 of 28 million patients (1.95%) had low CG (defined as <18 g/L) (Alfego, D., et al. 2025. *J. Hum. Immun.* Abstract 204. https://doi.org/10.70962/cis2025abstract.204). However, only a small minority of those patients had further relevant laboratory evaluations within the year following low CG. Data from a longitudinal analysis of low CG and a CVID ICD-10 code on any test ordered from Labcorp between 2012 and 2023 were also presented (Alfego, D., et al. 2025. *J. Hum. Immun.* Abstract 204. https://doi.org/10.70962/cis2025abstract.204). In total, 3,426 patients were identified as having an ICD-10 code for CVID at some point after having a low CG level (<18 g/L); the median number of days between these events was 1,089 (466–1,827; 3 years). These data illustrate that laboratory investigation of low CG would accelerate the diagnosis of CVID (Alfego, D., et al. 2025. *J. Hum. Immun.* Abstract 204. https://doi.org/10.70962/cis2025abstract.204).

**Figure 1.**
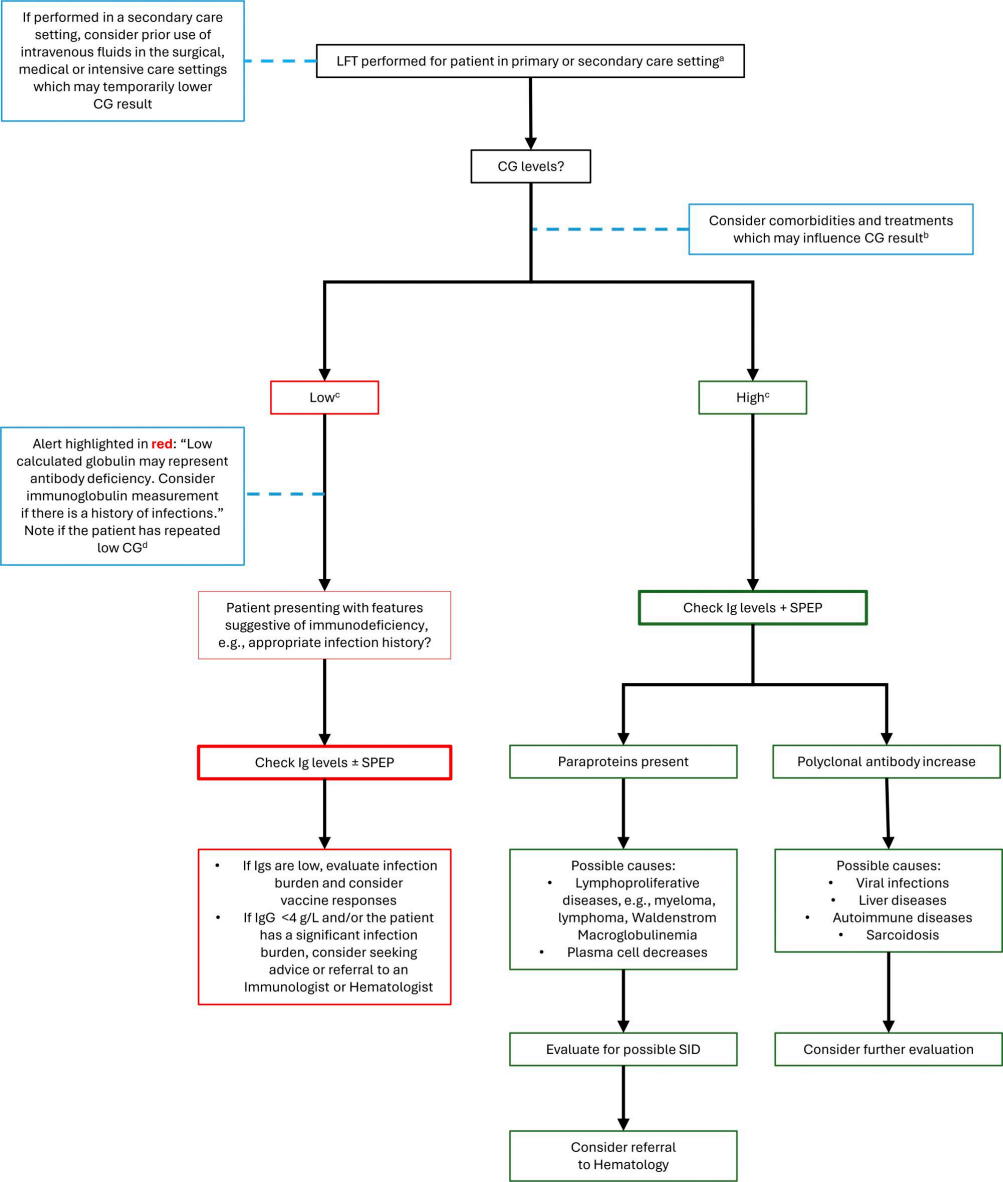
**Flowchart to guide CG screening and downstream testing** (10, 16, 17, 52, 55, 56, 57). ^a^Common manifestations of immunodeficiency include infections, inflammation, autoimmunity, and allergy. ^b^Comorbidities may include nephrotic syndrome, hepatic insufficiency, hyperlipoproteinemia, metastatic malignancy, and iron deficiency anemia. Treatments may include plasma exchange and FcRn inhibitors. ^c^Low and high CG action/cut-off levels range from <18 to 23 g/L and >33 g/L, respectively. Low CG levels may be indicative of a primary or secondary antibody deficiency; high CG levels may indicate lymphoproliferative disease, e.g., myeloma, viral infections, or autoimmune diseases (17). ^d^Serial low CG results may improve sensitivity and specificity for non-transient reductions in Ig. CT, computed tomography; IgG, immunoglobulin G; SPEP, serum protein electrophoresis; CG, calculated globulin; FcRn, neonatal Fc receptor; Ig, immunoglobulin; LFT, liver function test; SID, secondary immunodeficiency.

CG may be useful as an opportunistic screening prompt (or to monitor a CG trend) in patients receiving B cell depleting therapies or other immunosuppressive regimens, in whom secondary hypogammaglobulinemia can develop and persist long after treatment (4, 6, 53, 54). As these patients commonly undergo repeated routine biochemical monitoring for their underlying disease and treatment safety, CG can provide a low-burden signal and trend that also informs when formal Ig measurement should be considered—especially where systematic IgG surveillance is not embedded in routine pathways. Where appropriate, functional antibody testing may be considered following a confirmatory abnormal IgG result (Fig. 1).

### The cost of CG screening

Determining CG is inexpensive. Indeed, if total protein and albumin are routinely measured as part of the LFT profile, no further analyses are required, and there is no additional cost above those of determining LFTs—it is simply calculated from existing data (10). In contrast to NBS, which screens at a single time point, LFTs are often repeatedly performed over time, especially in patients who have ongoing symptoms, allowing trends in CG and, by implication, Ig, to be determined. Furthermore, data presented at the 2025 CIS Annual Meeting demonstrated that in the US in 2023, over 28 million patients had both albumin and total protein results as per Labcorp’s testing data (Alfego, D., et al. 2025. *J. Hum. Immun.* Abstract 204. https://doi.org/10.70962/cis2025abstract.204). Even if total protein is not included as a standard in the LFT profile, it is inexpensive to include (£0.07, US$0.11, €0.08 per test) (17). CG also fulfills the World Health Organization (WHO) criteria for a screening test (17, 58, 59). Consideration might be given as to whether CG screening could be designated as an essential test by the WHO, and more action is needed to support wider implementation of CG screening into routine practice.

## Implementation of CG in clinical practice

### Physician education and protocols

For implementation into routine clinical practice, primary and secondary care physicians need to understand the strengths and weaknesses of CG screening, and how to interpret the results. The way in which the CG results are conveyed to physicians is key. A clear explanation and interpretation of the test results and suggesting appropriate action (e.g., second-line confirmatory tests), especially if features include the manifestations of immunodeficiency, can be provided. This could help physicians (particularly primary care physicians) with downstream testing and onward specialist referral if antibody deficiency is confirmed, improving patient outcomes by facilitating early diagnosis and shortening the diagnostic odyssey (10, 17). These physician aids could be provided as automated electronic laboratory comments or notes with the test results (17), or what is known in the US as a clinical decision support alerting system (60). The General Practitioners Laboratory Management Committee Liaison Group in Wales selected the following comment for patients with low CG levels: “Low calculated globulin may represent antibody deficiency. Consider immunoglobulin measurement if there is a history of infections” (10, 17). As this comment alone may be overlooked or the results misinterpreted as being normal among the many other normal results received by busy clinicians, the result should also be flagged as abnormal in another way, such as highlighting it in red. However, health systems should consider monitoring alert exposure and develop and implement strategies to mitigate negative impacts, such as alert fatigue (physician unresponsiveness caused by cumulative exposure to alerts) that can contribute to delayed or missed diagnosis (60).

Jolles et al. proposed a method utilizing CG to assist in the diagnosis of patients with unsuspected immunodeficiency and to avoid diagnostic delays (17). They defined low CG as <18 g/L using the BCG method for albumin, which corresponded to IgG levels at the lower limit of their normal range (6–16 g/L). If a primary care physician requested an LFT test and CG was found to be low, the physician should be automatically alerted, and further action suggested in silico using the General Practitioners Laboratory Management Committee Liaison Group–approved wording (17). If the requesting physician is in secondary care, they have also suggested adding a caveat that the use of IV fluids in the surgical, medical, or intensive care settings may cause a temporarily low CG result. Furthermore, the location of the patient and sample are identified within the pathology management system, enabling bespoke comments for locations where, for example, IV fluid use was likely, such as intensive care or post-operative surgery wards. If further testing confirmed an antibody deficiency, the patient could then be referred to a specialist, e.g., an immunologist and/or hematologist, who could investigate the immunodeficiency, its potential cause, and initiate an appropriate management plan.

Holding et al. developed a similar screening protocol to facilitate the investigation of patients with low CG levels without delaying the biochemical results upon which the suspicion of hypogammaglobulinemia was based (11). Using an algorithm, the results from patients with CG <18 g/L (albumin measured using the BCP method) ≥10 years of age (children 9–10 years of age have CG levels approximating those in adults [17]) were referred to a clinical immunologist for assessment after exclusion of oncology sources (11). Between April 2007 and December 2012, 12 new patients with CVID were identified using this method. When these criteria were retrospectively applied to 5 patients with established CVID who had a median diagnostic delay of 4 years (2–30 years), the algorithm could have reduced the diagnostic delay by a median of 3.5 years (2 mo–18 years) (11).

Future developments could include the recognition in silico of serial low CG results, rather than a single result, to improve sensitivity and specificity for non-transient reductions in Ig, and the inclusion of CG into the Jeffrey Modell Foundation AI Software for Primary Immunodeficiency Recognition, Intervention, and Tracking analyzer (33). Educational initiatives will be fundamental resources for clinicians to understand the value of CG and what the results mean. CME is an essential tool to keep medical professionals up to date and informed of best practices (61). CME modules that cover CG screening to identify patients with unsuspected immunodeficiencies will be important to ensure that healthcare professionals are appropriately informed. It is anticipated that most patients may be identified at the primary care level; thus, clear instructions regarding next steps will be required if a patient has a CG indicative of immunodeficiency. These may include checking for serial low CG results and the patient’s clinical history, indicating when to order an IgG and other downstream tests, and importantly the criteria to liaise with, or refer the patient to, the immunology department and/or hematology department if an underlying hematologic malignancy is suspected. Physicians must be aware that where a low CG result prompts Ig measurement, but IgG is within the reference range, a single result may reflect transient factors (e.g., dilution from IV fluids or acute illness, as Igs can act as nonspecific acute phase proteins and decrease CG sensitivity). As Ig levels are often borderline in this setting, it is important to consider the overall trend in CG levels; if the trend is downward, then there is a likelihood of Igs also trending downwards, and repeated Ig monitoring may be considered. Low CG may also be associated with borderline normal Ig, which can be relevant if future immunosuppression or B cell ablation is required, as borderline Ig levels will more frequently and more quickly result in low Igs.

### Logistics

The logistics required to incorporate CG screening in routine clinical practice include the collaboration and buy-in of local laboratory services (usually the biochemistry department). However, approaches may vary depending upon the level of public versus private testing within differing healthcare systems, as expenses related to the supply of laboratory tests can account for 15–40% of the operational costs of a clinical laboratory (62). Due to its relatively low cost and simplicity, CG screening could help to improve the pathway of immunodeficiency diagnoses with antibody deficiency. Ig tests and other downstream assays have cost implications; hence, the sensitivity and specificity of CG screening and the clinician carefully considering if the clinical picture supports further testing are important. The magnitude of additional testing costs is influenced by the applied action threshold and the underlying patient population, which determines the positive predictive value (PPV) for low IgG. For example, in a large retrospective cohort (*n* = 4,381), the PPV for identifying low IgG among those with low CG was 82.5%, implying that 17.5% of follow-on IgG measurements will not confirm hypogammaglobulinemia despite a low CG result (Table 1) (10). This trade-off underscores the importance of implementing CG within a structured pathway to enable clinically led follow-on investigations while still enabling earlier identification of antibody deficiencies. Targeted testing for patients who are likely to have an abnormal IgG level will be more focused and thus more cost-effective than either untargeted/opportunistic testing or not making the diagnosis early, which can lead to the development of end-organ damage such as bronchiectasis (19). Furthermore, inclusion of comments to indicate abnormal CG results such as “consider immunoglobulins if there is a history of infections” (Fig. 1) will allow physicians to choose follow-on testing if deemed appropriate, which would help reduce unnecessary referrals and downstream additional costs.

Population-specific CG cutoffs of <18–20 g/L (using BCG to measure albumin) are based on data from several international studies in adults (Table 1) (5, 10, 17, 50). Population-specific CG cutoffs using the BCP method to measure albumin have also been identified (Table 1) (17). Applying these cutoffs will allow accurate interpretation of CG results and guide next steps, with the caveat that local laboratory adjustments may be needed (for example, if BCP is being used to measure albumin).

Multidisciplinary collaboration among diagnostic laboratories, primary and secondary care clinicians, patient organizations, medical societies, AI developers, and hospitals is required to optimize the potential benefits of CG screening.

### Successful implementation into routine practice

Due to the simplicity of determining CG and its affordability, CG can be widely implemented and not restricted to major hospitals or specialist services. CG is particularly suited to the primary care setting where most patients with undiagnosed immunodeficiencies will present first, and LFTs are also frequently performed. The use of IV fluids, which may impact the sensitivity and specificity of CG, is also much less likely in the primary care setting (17). These properties also make CG appropriate for use in resource-poor countries and communities. There is growing interest in using CG to screen for patients with immunodeficiencies among patient organizations, clinicians, and laboratory testing companies across multiple countries. Experts brought together to anticipate pivotal upcoming challenges and opportunities regarding PID at the second Global Multi-stakeholders Summit held by the International Patient Organization for PIDs support the universal use of CG screening to identify patients with unsuspected immunodeficiencies early (63). They also support the use of an automated flagging system with clear indicators for referring patients to a specialist (63).

With the support of the Brazilian Society of Clinical Pathology; the Brazilian Association of Asthma, Allergy, and Immunology; and the Brazilian Society of Pediatrics, automated CG calculations are being incorporated into routine practice when performing total protein and albumin measurements in major diagnostic laboratories in Brazil (52). In addition, following the establishment of lower action levels for both the BCG and BCP methodologies, national CG screening has also been introduced across National Health Service Biochemistry laboratories in Wales (17).

## Limitations and challenges of CG screening

While there is a linear relationship between CG and IgG (52; Alfego, D., et al. 2025. *J. Hum. Immun.* Abstract 204. https://doi.org/10.70962/cis2025abstract.204), an abnormal CG value may not wholly reflect an abnormal Ig level and vice versa, as there are other proteins present in the globulin fraction and other factors can affect globulin and/or albumin levels. For example, nephrotic syndrome and hepatic insufficiency can alter/lower serum albumin levels (and subsequently reduce the sensitivity of CG), while hyperlipoproteinemia, metastatic malignancy, and iron deficiency anemia can alter globulin levels (52, 64). Inflammatory disorders and chronic infections may cause significant increases in acute phase proteins, masking low CG levels in patients who may be immunodeficient (10, 52, 65). In addition, as CG levels reflect total Ig, elevated IgA and/or IgM levels (due to polyclonal or clonal causes) may mask low IgG (10). The clinical and laboratory context in which CG results are interpreted is important (e.g., acute inflammation with an elevated C-reactive protein, liver disease associated with abnormal transaminases and reduced albumin production, and renal loss of albumin based on renal function and urinalysis), and physicians should be aware that a low albumin associated with liver and renal conditions can decrease the sensitivity of CG to identify antibody deficiency. A flow diagram (e.g., Fig. 1) may be considered to help physicians identify these patients and inform accurate interpretation of CG results.

CG may also fail to identify patients with particular PIDs, for example, in specific antibody deficiency, who produce adequate IgG levels, but fail to mount a functional antibody response to pathogens or vaccinations (52). Physicians should also be aware that CG values may be affected by the clinical setting; for example, the administration of IV fluids in surgical, medical, or intensive care settings may cause a transient decrease in CG (17). CG levels can also be affected by treatments such as plasmapheresis and some neonatal Fc receptor (FcRn) inhibitors (17, 66).

### Establishing cut-off levels

While it would be of interest to validate country-specific “normal” CG levels to benchmark low and high thresholds, there has been a convergence of method-specific cutoffs in adults across a number of international studies, with population-specific cutoffs of <18–20 g/L (Table 1) (5, 10, 17, 50). However, local validation of these values remains important. Applying these cutoffs will allow accurate interpretation of CG results. Physicians would benefit from educational support in not only understanding how to interpret the results, but also the limitations of the test, how this may impact diagnosis and understanding that the results should not be interpreted in isolation; several low CG results over time or a falling trend can be helpful, and further confirmatory testing should be carried out if immunodeficiency is suspected. Guidance for physicians on downstream testing and the protean manifestations of immunodeficiency will enhance the likelihood of an antibody deficiency diagnosis. Such guidance may take the form of a linked flowchart or toolkit, enabling successful translation and implementation into clinical and laboratory practice (Fig. 1) (10, 16, 17, 52, 55, 56, 57).

There is a need for age-specific CG thresholds to identify pediatric patients (<10 years of age) with immunodeficiencies. Both IgG and CG levels vary according to age groups, highlighting the need for different CG cutoff/action values depending on the patient’s age (16, 52, 65). Data are available regarding meaningful CG thresholds in children from three studies, but these data are not as clear-cut as those from international studies in adults (16, 52, 65).

### Increased diagnoses and demands on treatment

CG is of less utility in establishing a primary diagnosis in patients in whom an antibody deficiency is already suspected, as directly measuring IgG should yield definitive results, but rather more as an opportunistic (and often repeated) screening for patients undergoing albumin and total protein measurements (LFTs) as part of their routine care for other diagnoses and where antibody deficiency has not yet been considered (52).

The use of CG screening in clinical practice will result in more patients being diagnosed with PAD or SAD (hypogammaglobulinemia) and subsequently increase demand for patient assessments and interventions, including infection risk mitigation strategies such as prophylactic antibiotics, vaccinations, optimization of comorbid conditions, pathogen-exposure mitigation, and IgRT (67, 68, 69). The global demand for IgRT has been shown to increase annually by 6–8%, with higher rates in emerging countries due to lower initial consumption levels (67). In a retrospective analysis of the UK National Immunoglobulin Database and the UKPID registry, 3,000 patients who had received IgRT were identified, two-thirds of whom (61%) had antibody disorders (70). The IgRT supply is reliant on global donations of plasma (70), 65% of which is donated in the US (67). Moreover, the increased demand for IgRT to treat immunodeficiencies may have a knock-on effect on other specialties that use Ig, such as neurologists who treat autoimmune conditions, for example, chronic inflammatory demyelinating polyneuropathy and multifocal motor neuropathy (70). A global effort will be required to increase plasma donations to meet increasing demand (67) alongside improving manufacturing processes to increase the yield of Ig from every donation (71). Of note, there are some areas in which demand is decreasing due to the use of new therapies (e.g., FcRn inhibitors and B cell targeting treatments), replacing the use of Ig in some immunomodulatory settings (67).

Furthermore, the increased use of CG may result in a higher demand for access to physician specialties such as clinical immunologists. Several approaches may be considered to limit potentially unnecessary referrals, such as the use of defined and validated cut-off levels tailored to the specific population and comments on laboratory results which can provide direction on the next steps, including additional testing such as functional antibodies (Fig. 1). Observation of several low CG results or a declining trend may also assist in refining prioritization of cases requiring further review and/or referral. Discussions with other specialties may also be helpful where an antibody deficiency may arise due to the underlying disease (e.g., a hematologic malignancy) or therapy, such as chimeric antigen receptor T-cell therapy (CAR T) or chemotherapy, prior to the introduction of CG screening.

Finally, while much of the existing evidence for CG screening is derived from retrospective cohorts, larger multicenter prospective studies in more ethnically diverse populations will be valuable to further refine diagnostic performance, action thresholds, and evaluate the utility of low CG screening across different healthcare settings.

## Conclusion

CG is a simple, cost-effective and accessible screening test for immunodeficiencies that is currently underutilized in clinical practice. Unlike conventional screening (e.g., NBS), repeated CG screening allows for the assessment of Ig trends over time. There is a convincing argument to implement CG screening more widely, along with method-specific cut-off levels for adults, agreed automated comments, and downstream testing and referral criteria. CG is a viable screening method for antibody deficiency in PID and SID in both adult and pediatric patients (5, 16, 17, 50, 52; Alfego, D., et al. 2025. *J. Hum. Immun.* Abstract 204. https://doi.org/10.70962/cis2025abstract.204; Jolles, S., and C. Poli, personal communication). Recently, in a large retrospective study of 28 million tests in the US, CG screening has been shown to be associated with a potential reduction in the delay in the diagnosis of PID (specifically CVID) (11), from 6 to 8 years after symptoms first appear without CG to a median of 3 years following detection of the first low CG levels, with potential savings of approximately US$68 million (Alfego, D., et al. 2025. *J. Hum. Immun.* Abstract 204. https://doi.org/10.70962/cis2025abstract.204). CG screening is also being incorporated into various AI approaches to facilitate early diagnosis. The widespread implementation and uptake of CG screening for antibody deficiencies requires a collaborative effort from clinicians, diagnostic laboratories, patient organizations, and policy makers across a range of healthcare settings. CG screening has the potential to improve the rate of diagnosis and treatment of both PAD and SADs.
